# Horticultural performance and QTL mapping of snap bean (*Phaseolus vulgaris* L.) populations with organic and conventional breeding histories

**DOI:** 10.3389/fpls.2025.1533039

**Published:** 2025-05-19

**Authors:** Hayley E. P. Richardson, Ryan M. King, Joel Davis, James R. Myers

**Affiliations:** ^1^ Department of Botany and Plant Pathology, Oregon State University, Corvallis, OR, United States; ^2^ National Clonal Germplasm Repository, Agricultural Research Service, U.S. Department of Agriculture, Corvallis, OR, United States; ^3^ Department of Horticulture, Oregon State University, Corvallis, OR, United States

**Keywords:** seed color, seed weight, germination, recombinant inbred lines, linkage map, root traits, agronomic traits

## Abstract

**Introduction:**

Improving crop cultivars for use on organic farms is pertinent, as current elite germplasm is less resilient within the more variable context of organic farm environments. Although a growing number of studies have focused on organic plant breeding in cereal crops, very few have focused on vegetable crops, especially those such as snap beans (*Phaseolus vulgaris*) that are grown for both fresh market and processing use.

**Methods:**

We developed four populations of recombinant inbred lines under parallel organic and conventional management; utilizing these populations, we explored how historic breeding history influences the performance of snap bean progeny.

**Results:**

We identified significant increases in germination speed and rate, suggesting that beans bred within an organic production environment are more resilient to early-season stressors without support of chemical interventions. We also found that root branching density increased among organically-bred bean families, while root disease decreased in both the organically-bred bean families and the populations with ‘OR5630’ × ‘Black Valentine’ parentage. After developing linkage maps for each of our four populations, we identified QTL associated with days to germination, early-season vigor, root morphology, disease, days to flowering, and seed weight.

**Discussion:**

This study lays the groundwork for improving snap bean germplasm for performance in organic systems by tracking the microevolutions created through long-term selection under organic or conventional management (i.e., breeding history). By understanding these shifts, plant breeders will begin to build a toolbox of genetic information that they can leverage in modern breeding work for organic crop cultivars.

## 1 Introduction

Over the past two decades, elevated public demand for organically produced foods has more than doubled the retail sales of organic food within the United States (USDA ERS, 2024). Consumer interest in health, wellness, and ecological sustainability drives much of this demand (Lee and Yun, 2015). Studies have found that organic crops have higher quantities of antioxidants, lower concentrations of heavy metals, and reduced pesticide residues compared with conventional crops (Barański et al., 2014). Other studies determined that organic agriculture requires fewer non-renewable resources, reduces nonpoint source pollution from agrochemicals, and provides producers with opportunities for higher profit margins (Badgley et al., 2007; Bengtsson et al., 2005; Shreck et al., 2006).

Despite demand, the persistent yield gap between organic and conventional production limits the viability of organic agriculture at scale. Meta-analyses attempting to capture the yield gap found that organic yields average 20-25% lower than conventional (Kniss et al., 2016; Ponisio et al., 2015; Seufert et al., 2012). Though these analyses provide a high-level view of yield gap scale, each study notes high variation across several parameters, including crop type (i.e., cereal, legume, forage, etc.), geographic location, and input intensity of the operations (i.e., high vs. low fertilizer applications). Across all crops with available data, organic fruits and vegetables have the greatest yield gaps, with yields falling 23-62% lower than conventional counterparts (Kniss et al., 2016). This is likely due to the increased impact of insects and pathogens on the visual marketability of these crops, which are frequently sold fresh or direct-to-consumers.

The amount of plant-available nitrogen and the scale of perennial weed pressure are consistently identified as the primary driving forces underlying the yield gap (Knapp and van der Heijden, 2018; Kravchenko et al., 2017). Agronomic solutions to these challenges are under continuous development (Cabrera-Pérez et al., 2023; Reimer et al., 2023; Rowland et al., 2023); yet, incorporating these techniques on-farm may be technically or financially unfeasible for growers. Thus, the breeding and development of new cultivars that are well-suited to organic farm conditions emerge as critical “low-cost, high-impact” solutions to the yield gap.

Unlike conventional crops, organic crops must be able to cope with highly dynamic environments without the buffer of supportive chemical inputs. Plasticity is widely observed in cultivated and wild plant populations, and can be considered a distinct quantitative trait that provides some individuals with the capacity to more rapidly adapt to challenges in their environment. Plasticity may be further characterized as genotype-by-environment (G × E) interactions, wherein the capacity for adaptive plasticity of a given phenotype varies among genotypes (Laitinen and Nikoloski, 2019). While historic plant breeding has attempted to eliminate phenotypic plasticity by controlling the growing environment, the current and future implications of anthropogenic climate change drive demand for cultivars that can exploit adaptive plasticity in unpredictable environmental conditions (Brooker et al., 2022). Similarly, in highly dynamic organic environments, plasticity is a valuable trait that organic breeding strategies should seek to leverage.

The impact of G × E interactions on the performance of a genotype in specific test environments, and the performance of a genotype in the target population environments, underpins the case for developing organic cultivars within organic environments (Allen et al., 1978). When the correlation between performance in a test environment and performance in a target population of environments, referred to as *r*, is high, the testing environment used is considered typical of the target population of environments (TPEs). When correlation values are low the environments are considered unique in comparison with the TPE. Conventional breeding strategies have historically operated under the understanding that heritability may decrease in stressed environments, thus direct selection should not be carried out under ambient field stress. Instead conventional breeding strategies have relied on a combined method of direct selection for yield under optimal environments and indirect selection of physiological traits under simulated stress conditions (Blum and Jordan, 1985). However, other breeding strategies maintain that heritability is not more variable under stressed conditions, and a breeding approach that optimizes *r* through direct selection within these environments may also be effective, or preferable in some cases (Allen et al., 1978; Ceccarelli, 1989).

In the context of organic agriculture, several studies have identified improvements in characteristics like marketable weight (i.e., yield), competition against weeds, and disease resistance in cultivars selected under organic management compared to their conventional counterparts. To date, most of this work has materialized within cereal crop breeding programs (Murphy et al., 2007; Schneider et al., 2024; Wolfe et al., 2008), excepting some recent work in potato, carrot, and tomato (Blossei et al., 2024; Colley et al., 2025; Lopez et al., 2024). Other vegetable crops, including common beans, have only been the target of observational studies within organic environments (Ozaktan et al., 2023).

Common bean (*Phaseolus vulgaris* L.) was domesticated for use as a crop approximately 8,000 years ago in two separate regions: the Andes and Middle America (Wallace et al., 2018). Snap beans are the form of common beans grown for their stringless, low-fiber vegetable pods; a category that includes green beans, wax beans, and flat-podded Romano beans. These may be harvested for both fresh markets and processing markets, with a distinct crop ideotype for each target market. The processing bean ideotype includes an upright growing habit, suitability for mechanical harvest, palatable appearance after processing, appropriate sieve size at harvest, round pods, concentrated pod set, and white seeds. The fresh market ideotype differs in that oval pods are acceptable, the pods may have higher fiber content for straighter and smoother pods, and pubescence to provide cushioning and prevent damage during handling and transport. Colored seeds may also be acceptable in some markets.

Within the Pacific Northwest, and western Oregon specifically, snap bean production thrives as a product of low disease pressure and irrigation capabilities. Cool, wet spring conditions that hamper crop establishment and early season performance are typical for snap bean production in the region. Factors such as darker seed color and thicker seed coat can improve germination within these challenging environments; however, these traits lie in conflict with processing industry preference for white-seeded cultivars. Within conventional systems, snap bean seeds are coated with fungicidal treatments before sowing, and a pre-emergence herbicide is frequently applied. These approaches eliminate the need for highly vigorous seedlings capable of outgrowing early pressures from pathogens, insects, and weeds. Furthermore, conventional farmers rely on synthetic fertilizers throughout the snap bean growth cycle, a practice which has been found to reduce nutrient-seeking growth in the root system (Duncan et al., 1960; Lammerts van Bueren et al., 2011). While historic breeding practices have met the demands of conventional growers, breeding exclusively within conventional, high-N input systems has resulted in the unintentional loss of essential plant functions, such as the inactivation of legume genes responsible for supporting mutualism with nitrogen-fixing soil bacteria (Toby et al., 2007). A loss of function in this mutualism through the breeding process has minor implications in conventional fields where synthetic nitrogen is available, but can be devastating in organic fields that partially rely on nitrogen fixation. This example emphasizes the need to optimize *r* when developing new cultivars for a target environment, like organic production systems.

Our work was guided by the hypothesis that selection under organic and conventional management leads to divergent phenotypic expression and changes in the associated genetic architecture. To explore this, we split two populations of snap beans into two target environments, organic and conventional, and allowed natural selection in each system to enact pressure on the sub-populations. We subsequently evaluated sub-populations in both systems to test whether breeding under contrasting environments produced genotypes with distinct performance, levels of plasticity, and adaptive traits relevant to each environment. Additionally, we explored segregation distortion and QTL differences to determine whether differential selection drove measurable shifts at the genetic level.

## 2 Materials and methods

### 2.1 Population development

We developed four populations of recombinant inbred lines (RILs) of snap beans. Two populations were bred under organic management and two were bred under conventional management. We chose parental cultivars for these crosses in a manner that pairs a modern, elite snap bean (Hystyle and OR5630) with an older cultivar with good performance in organic and low-input conditions (Provider and Black Valentine), as described previously (Park et al., 2023). The elite parents, Hystyle and OR5630, were selected due to their current use by commercial farmers for the processing industry. Previous work by Kraemer and Myers (2014) identified Provider and Black Valentine as showing positive yield responses following inoculation with *Rhizobia*, in lieu of synthetic nitrogen. The ability for these cultivars to yield well without conventional fertilizer motivated their use as parents in this study. **Table 1** includes details on the origin, release date, and genetic origin. **Supplementary Table 1** includes additional phenotypic data on parental cultivars.

**Table 1:** Characteristics of the four snap bean cultivars used to create the research populations.

Parent Cultivar	Origin	Release Date	Structure Analysis (K = 2)		Structure Analysis (K = 8)				
			Middle American	Andean	Group 3 Bush blue lake	Group 4 European extra fine	Group 5 Andean landrace & Romano	Group 6 Andean II	Group 7 Andean I
Hystyle	Harris Moran Seed Company	1985	0.000	1.000	0.000	0.000	0.000	0.623	0.377
Provider	USDA SE Veg. Breed. Lab.	1966	0.024	0.976	0.000	0.040	0.344	0.579	0.000
OR 5630	Oregon State University	2005	0.437	0.563	1.000	0.000	0.000	0.000	0.000
Black Valentine	Peter Henderson & Co.	~1897	0.000	1.000	0.000	0.000	0.238	0.762	0.000

Structure analysis data from Wallace et al. (2018) shows genetic relationships of the cultivars, including K = 2 clusters associated with Middle American or Andean origin, and K = 8 clustering showing genotypic distribution across five of the eight groups (Groups 1, 2 and 8 omitted because parents showed no genetic affinity for those groups). Additional phenotypic data can be found in **Supplementary Table 1**.

Cultivars were identified as strong performers in either conventional commercial production for the processing market or the home garden environment.

Following the initial crosses, we grew the F_1_ generations in the field at the Vegetable Research Farm in Corvallis, OR. In the F_2_ generation, 500 seeds from each cross were grown under either organic or conventional management. The four populations were further developed after this split through a series of generation advances described in **Supplementary Table 2**. As of the F_6_ generation, each population is composed of approximately 90 immortalized and inbred lines.

Our breeding method applied natural selection pressure on each of the four breeding populations, creating two sets of populations that can be used to partition breeding management system effects and population genome structure. To our knowledge, this is the only set of snap bean populations in existence designed to investigate questions related to breeding under organic management.

### 2.2 Study sites and experimental design

For this study, we grew and evaluated the four populations over three years: 2018, 2020, and 2021. The 2018 plots were planted at the Lewis Brown Farm, at latitude 44.555881°N and longitude 123.214569°W. The organically-bred populations (ORBV-O, HYPR-O) were grown in an organically-managed field, and the conventionally-bred populations (ORBV-C and HYPR-C) were planted in an adjacent conventionally-managed field. In 2020 and 2021, all four populations were planted into two adjacent fields at the Vegetable Research Farm at Oregon State University. The farm is located at latitude 44.571209°N and longitude 123.243261°W. Each year we managed one of the two adjacent fields conventionally and the other organically. Experimental design details can also be found in **Table 2**, along with a list of traits evaluated in each year.

**Table 2:** Experimental design for field plots grown including year, production system, field location, traits evaluated, and populations grown.

Year (Generation)	Traits Evaluated	Location	Production System	Populations Grown
2018 (F_6_)	Days to 50% Flowering, Seed Weight/Plant, 100 Seed Weight, Germination Percent	Lewis Brown Farm, Corvallis, OR	Organic	ORBV-O, HYPR-O
			Conventional	ORBV-C, HYPR-C
2020 (F_7_)	Germination Percent, Days to Germination, Seedling Vigor, 100 Seed Weight	Vegetable Research Farm, Corvallis, OR	Organic	ORBV-O, ORBV-C, HYPR-O, HYPR-C
			Conventional	ORBV-O, ORBV-C, HYPR-O, HYPR-C
2021 (F_8_)	Germination Percent, Days to Germination, Seedling Vigor, Taproot Diameter, Basal Root Diameters, Branching Density, Root Disease Rating	Vegetable Research Farm, Corvallis, OR	Organic	ORBV-O, ORBV-C, HYPR-O, HYPR-C
			Conventional	ORBV-O, ORBV-C, HYPR-O, HYPR-C

Each year, the research plots were planted in a randomized complete block design, with three replications of each family planted into every population block. Single family plots were 1.5 meters in length, with 2.5 cm in-row spacing of plots, and 76 cm between-row spacing. We planted 60 seeds in each plot. An early planting date, immediately after fields became workable, was selected in both years to maximize the early season pressure on seeds and seedlings. Conventional management included a pre-planting seed coat application of Captan fungicide, Dual-Magnum pre-emergence herbicide application, and a custom fertilizer blend (12-10–10 N-P-K) at a rate of 618 kg ha^-1^. Organic management included compost (Recology Organics, Aumsville, OR in 2020; on-farm compost in 2021) at a rate of 22.9 m ha^-2^ and Nutri-Rich chicken litter-based fertilizer (8-2–4 N-P-K) at a rate of 1,159 kg ha^-1^.

### 2.3 Field evaluations

In 2018, we collected data from the F_6_ generation on germination-related traits, flowering, seed color, and 100-seed weight. Germination traits included days to germination and germination percentage, as calculated from the number of emerged seedlings minus those that had characteristic lethal damage that limited their survival past the seedling stage. Days to flowering was determined in the 2018 plots by rating the plot when 50% of the plants had open flowers. Following harvest and a drying period, the 100-seed weight was collected from 100 seeds of each plot replicate.

In 2020 and 2021, we collected data on germination-related traits, 100-seed weight, seed color, and five root morphology traits. Germination and seed weight data were collected as described above. We collected root data on a random subset of 40 families within each population, in both the organic and conventional fields. Samples were collected for these 40 families across all three reps, within each production system. Samples were collected over a two-week period, beginning when at least 50% of plots had reached 50% flowering. The conventional field was sampled in the first week and the organic field was sampled in the second week. To sample, we selected one representative plant from the center of each plot that displayed typical morphology and in-row spacing for the plot. Plants were harvested by digging a 30 cm radius of soil surrounding the stem, as described by (Burridge et al., 2016). Excess soil and roots from surrounding plants were gently shaken loose prior to the removal of aboveground plant matter. Roots were cleaned by immersion in water and then evaluated for taproot and basal root diameters, branching density, and disease severity of bean root rot complex. Previous work at the Vegetable Research Farm has consistently found *Fusarium solani* to be the dominant causal agent of root rot disease; however, *Rhizoctonia solani* may also be present in the complex at this site (Cirak and Myers, 2021; Hagerty et al., 2015; Huster et al., 2021). Root diameters were measured in mm using calipers, approximately 1 cm below the attachment point to the hypocotyl. We rated disease severity on a 9-point hedonic scale, where 0 represents no lesions on roots and 9 represents an extremely high number of lesions. All data were collected by a single individual to ensure consistency.

### 2.4 DNA extractions and genotyping

We extracted DNA from plant material collected from young trifoliate leaves of each family in the four populations. Samples for the HYPR populations were collected from field-grown plants in the F_7_ generation, and ORBV population samples were collected from F_8_ seedlings grown in a greenhouse. We extracted DNA from the F_7_ samples using a modified cetyltrimethylammonium bromide (CTAB) protocol (Miklas et al., 1993). For the F_8_ samples, we extracted DNA using a modified extraction protocol for the E-Z 96 Plant DNA extraction kit (Omega BioTek, Norcross, GA, USA).

Samples were quantified on the BioTek Synergy 2 microplate reader and diluted to approximately uniform concentrations. Ten microliters of dilute samples were placed into 96-well plates, which were sent to the USDA-ARS Soybean Genomics and Improvement Laboratory at the Beltsville Agricultural Research Center in Beltsville, MD, for genotyping with the Illumina BARCBean12K beadchip (Song et al., 2015). Variant calls were confirmed using Illumina’s GenomeStudio v2.0.5 software.

### 2.5 Linkage and QTL mapping

*Genetic map construction*

Prior to creating the linkage maps, we examined and filtered raw SNP calls in Microsoft Excel 2019 to remove markers that were monomorphic or heterozygous in the parents. We then loaded these filtered and aligned data sets into JoinMap5 for further inspection and map construction (van Ooijen, 2016). SNPs were removed in an iterative process based on the number of missing allele calls (>27%), marker redundancy (based on similarity values of 1.00), high heterozygosity (>11.25%), and segregation distortion (*X^2^* > 9.67). Individuals with high heterozygosity were removed from each population, as these were suspected to have undergone accidental outcrossing. We estimated linkage groups in JoinMap5 using the linkage logarithm of odds (LOD) grouping parameter, with a threshold of 5.0 for significant pairwise linkages. Distance in centimorgans (cM) was determined using Kosambi’s mapping function and the maximum likelihood algorithm, with optimization parameters set to a chain length of 10,000 iterations, 1,000 iterations for multipoint estimation of recombination frequency, a burn-in chain length of 15,000, and otherwise default settings. The resulting maps were visually inspected for large intra-map gaps (>20cM) and markers showing high nearest neighbor stress values. Where necessary, markers were added and removed to generate final maps.

*Quantitative trait loci analysis*

Quantitative trait loci and associated LOD scores were identified using the R package *qtl2* (Broman et al., 2019). Prior to these calculations, we averaged the phenotypic data across replications to increase detection power. Using the function *scan1* and Haley-Knott regression, we identified flanking SNPs of sufficiently large QTL (Haley and Knott, 1992). We ran permutation tests to account for the possibility of false positives in QTL detection and to determine significance thresholds for QTL detection for each phenotypic trait. Additive effects of each QTL and the percent phenotypic variation explained, as represented by R^2^, were also calculated.

### 2.6 Segregation distortion

Utilizing physical locations of SNPs, allele frequencies, and the *X^2^* values generated in JoinMap5, we assessed segregation distortion within each population. We considered markers with a *X^2^* value above 5.99 distorted. Using the *chromoMap* package in R Studio, we constructed distortion maps with physical locations of SNPs aligned using the G19833, v2.1, reference genome of Andean origin (Anand and Rodriguez Lopez, 2022; Schmutz et al., 2014). Scaffolds lacking genome-aligned locations were removed from the maps.

### 2.7 Statistical analyses

All statistical analyses were completed in R Studio, using the *dplyr* package (Posit Team, 2024; R Core Team, 2023; Wickham et al., 2022). An analysis of variance (ANOVA) was conducted to test major experimental contrast factors (e.g., seed color, breeding history, parental cross, etc.), with mean square values reported in **supplementary material**. We then calculated estimated marginal means for each trait, developing a linear mixed model for each trait using the *lme4* and *emmeans* packages (Bates et al., 2024; Lenth et al., 2024). Data from each year and production system were analyzed separately. For each mixed model, seed color, cross, and breeding history were included as fixed effects and rep was a random effect. A second set of marginal means was calculated to explore the role of seed color within populations. In these models, the population factor was used as a fixed effect in lieu of the breeding history and cross factors, but seed color and rep remained the same. Tukey HSD tests were run for multi-year, multi-environment estimated marginal means.

To examine the strength and direction of associations between early season performance, root phenotypes, and seed characteristics, we ran a Spearman’s rank correlation analysis in R Studio and visualized the correlations using the *corrplot* package (Wei et al., 2024). The Spearman’s rank correlation allowed for the inclusion of ordinal seed color data in the analysis. Correlation coefficients were generated for each pairwise combination of traits. Due to the categorical nature of color-based data, we converted seed color to a numerical scale in which an absence of pigment (i.e., white) was associated with the value 0 and the presence of pigment (i.e., colored seed) was associated with the value 1.

Best linear unbiased predictions (BLUPs) were calculated for the estimation of variance components and heritability. The data for these analyses originated from the F_6_, F_7_, and F_8_ generations, and there were three replications for each trait within each environment. Data were classified as multi-year and multi-environment, single-year and multi-environment, or single-year and single-environment.

Narrow-sense heritability was estimated using the following formula for multi-environment heritability (Isik et al., 2017):


$${\text{h}}^{2}=\sigma {\text{F}}^{2}/(\sigma {\text{F}}^{2}+(\sigma {\text{FE}}^{2}/\text{s})+(\sigma \epsilon^{2}/\text{sr})).$$

For data collected in a single environment, the following formula was used to estimate narrow-sense heritability (Singh et al., 1993):


$${\text{h}}^{2}=\sigma {\text{F}}^{2}/(\sigma {\text{F}}^{2}+\sigma \epsilon^{2})$$

In both formulae, *σ*F^2^ is the estimated family-wise genotypic variance component, and *σ*_ε_^2^ is the estimated experimental error variance component. Additional terms for multi-environment data include: *σ*FE^2^ as the estimated family-by-environment (F × E) interaction variance component, s as the number of environments, and r as the number of replications in each environment. For multi-year and multi-environment data, we calculated the variance components for F × E interactions across years and production systems.

G × E interactions were explored for all traits collected in 2020 and 2021, including germination percentage, days to germination, seedling vigor, root diameters, root branching density, and disease resistance. Linear or generalized mixed models were used to calculate the population-wide estimated marginal means for each trait.

For the evaluation of G × E interactions in germination and vigor data, the following model was used:


$$Y_{ijkl}=\mu +P_{i}+S_{j}+{\left(P\times S\right)}_{ij}+C_{k}+(1|R/F_{l})+(1|Y_{m})+\in ijkl$$

where *Y_ijkl_* ​ is the observed trait, *μ* is the overall mean, *P_i_*, *S_j_*, and *C_k_* ​ are the fixed effects of population, production system, and seed coat color, respectively, *(P×S)_ij_* represents their interaction, (1∣*R/F_l_*) is the random effect of family nested within replication block, (1∣*Y_m_*) is the random effect of sampling year, and *∈_ijkl_* is the residual error.

For the evaluation of G × E interactions in root morphology and disease data, where only one year of data was collected, the following model was used:


$$Y_{ijkl}=\mu +P_{i}+S_{j}+{\left(P\times S\right)}_{ij}+C_{k}+(1|R/F_{l})+\in_{ijkl}$$

where *Y_ijkl_* ​ is the observed trait, *μ* is the overall mean, *P_i_*, *S_j_*, and *C_k_* ​ are the fixed effects of population, production system, and seed coat color, respectively, *(P×S)_ij_* represents their interaction, (1∣*R/F_l_*) is the random effect of family nested within replication block, and *∈_ijkl_* is the residual error. Significance of the G × E interactions in each model was then analyzed using the *joint_tests* function in the *emmeans* package to run Type III tests.

## 3 Results

### 3.1 Early-season performance varies according to breeding history and seed color

Utilizing the data collected from the F_6_-F_8_ generations, we explored the relationship between population factors and early season performance. Analysis of variance (ANOVA) indicated that each of the examined factors – seed color, breeding history, cross, production system, and the breeding history × system interaction – had a significant influence on days to germination and germination percentage across all years (p < 0.05 for all contrasts). These results, shown in **Supplementary Table 3**, motivated further exploration of these traits across the factor gradients. We generated estimated marginal means (EMMs) for all traits and populations within each year and production system (**Supplementary Table 4**), as well as for the white-seeded and pigmented seed families of each population within each year and production system (**Supplementary Table 5**).

**Figure 1:**
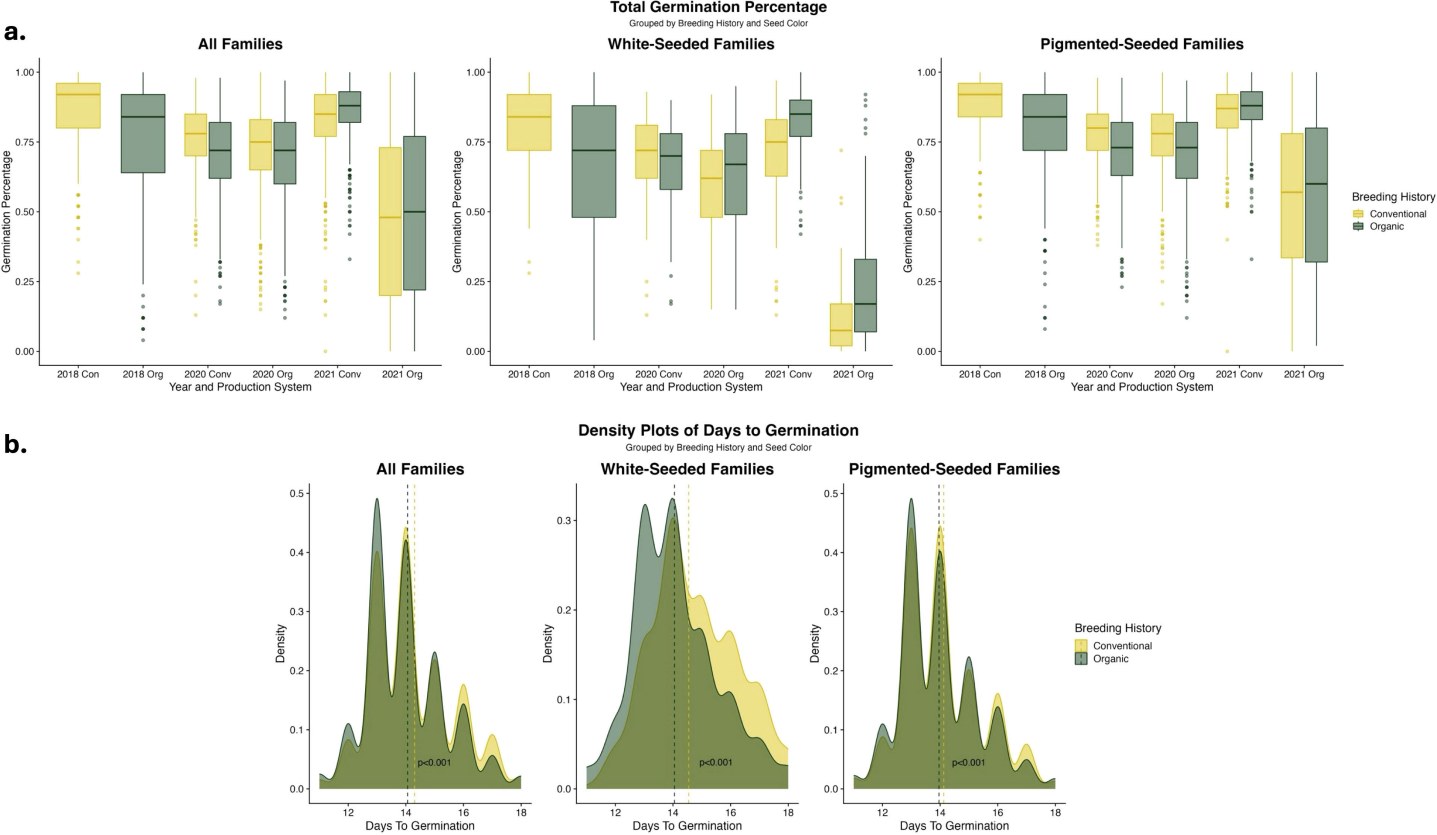
**(a)** Boxplots of germination percentage of F_6_-F_8_ families categorized by seed color and breeding histories, analyzed within each year and production system. Density plot color indicates population data from each breeding history or the overlap of data. **(b)** Density plots of days to germination for F_7_ and F_8_ families categorized by seed color and breeding histories, analyzed across experimental years, populations, and production systems. Vertical lines indicate the estimated marginal means for each breeding history group within each seed color category. Significance values are derived from Tukey HSD tests. Days to germination data was not collected on the F_6_ generation in 2018.

Notable year-to-year variation in germination percentage was identified, with particularly low germination in the 2021 organic treatment, shown in **Figure 1a**. Germination was lower for the organically-bred families in 2018, although it should be noted that the fields were not planted reciprocally during this year (i.e., the organically-bred families were only grown in organic fields). There were varying trends in germination rate across all families and breeding histories in 2020 and 2021; however, there were consistent increases in the germination percentage of white-seeded families with an organic breeding history. Visualizing the days to germination across breeding histories revealed a trend towards faster germination in the organically-bred, white-seeded families, shown in **Figure 1b**.

EMMs for the germination traits, shown in **Table 3**, indicate clear trends of higher germination rates in the organically-bred, white-seeded families in 2020 and 2021 within the organic fields. When the EMMs were calculated across the data from all of the fields in 2020 and 2021, the organically-bred, white-seeded families had significantly higher (α < 0.05) germination percentage (0.598 ± 0.083) than their conventionally-bred counterparts (0.558 ± 0.083). The multi-year, multi-environment average of days to germination was significantly lower (α < 0.05) for the organically-bred, white-seeded families (14.1 ± 0.83) than for the conventionally-bred, white-seeded families (14.5 ± 0.83), indicating faster germination.

**Table 3:** Estimated marginal means (EMMs) with standard errors for germination percentage and days to germination of snap bean families with organic and conventional breeding histories.

Breeding History		2018		2020 Organic Field		2020 Conventional Field		2021 Organic Field		2021 Conventional Field		2020 and 2021 - All Fields	
		Days to Germination	Germination Percentage	Days to Germination	Germination Percentage	Days to Germination	Germination Percentage	Days to Germination	Germination Percentage	Days to Germination	Germination Percentage	Days to Germination	Germination Percentage
All Families	Organic	NA	0.741 ±0.009^a^	13.9 ±0.059^a^	0.661 ±0.011^a^	15.7 ±0.055^a^	0.686 ±0.008^a^	12.9 ±0.038^a^	0.392 ±0.012^a^	13.7 ±0.039^a^	0.835 ±0.008^a^	14.10 ±0.72^a^	0.645 ±0.028^a^
	Conventional	NA	0.844 ±0.009^b^	14.1 ±0.06^b^	0.690 ±0.011^b^	16 ±0.056^b^	0.745 ±0.008^b^	13.1 ±0.04^b^	0.351 ±0.012^b^	13.9 ±0.039^b^	0.791 ±0.008^b^	14.30 ±0.72^b^	0.647 ±0.028^a^
White- Seeded Families	Organic	NA	0.684 ±0.021^a^	14.0 ±0.092^a^	0.644 ±0.014^a^	0.08 ^a^	0.676 ±0.013^a^	13.0 ±0.11^a^	0.243 ±0.016^a^	13.5 ±0.097^a^	0.823 ±0.014^a^	14.1 ±0.83^a^	0.598 ±0.083^a^
	Conventional	NA	0.838 ±0.022^b^	14.6 ±0.082^b^	0.626 ±0.016^a^	16.2 ±0.09 ^b^ 15.8 ±	0.717 ±0.014^b^	13.3 ±0.13^a^	0.141 ±0.018^b^	14.0 ±0.10^b^	0.725 ±0.015^b^	14.5 ±0.83^b^	0.558 ±0.083^b^
Pigmented Seed Families	Organic	NA	0.793 ±0.008^a^	13.7 ±0.063^a^	0.700 ±0.011^a^	15.5 ±0.05^a^	0.707 ±0.007^a^	12.8 ±0.034^a^	0.567 ±0.013^a^	13.8 ±0.038^a^	0.871 ±0.009^a^	14.0 ±0.69^a^	0.711 ±0.014^a^
	Conventional	NA	0.886 ±0.007^b^	13.8 ±0.063^a^	0.746 ±0.011^b^	15.9 ±0.051^b^	0.774 ±0.007^b^	12.9 ±0.034^b^	0.545 ±0.013^a^	13.9 ±0.038^b^	0.847 ±0.009^b^	14.1 ±0.69^b^	0.728 ±0.014^b^

Means followed by different superscript letters indicate significant pairwise differences between breeding histories, based on Tukey’s HSD test. (α < 0.05).

Narrow-sense heritability, shown in **Table 4**, was high for the early-season traits. Heritability for days to germination across all years and environments ranged from 0.51 to 0.75 across populations. The range was lower, at 0.36 to 0.66, for germination percentage. Early-season vigor had the highest heritability, ranging from 0.78 to 0.83. We identified moderate to high heritability for these traits within the organic systems, ranging from 0.30 to 0.71, and low to high heritability in the conventional systems, ranging from 0.12 to 0.80.

**Table 4:** Narrow-sense heritability of traits collected in multiple years (2020 and 2021) and multiple environments, for each population.

Production System	Trait	Narrow-Sense Heritability			
		ORBV-O	ORBV-C	HYPR-O	HYPR-C
Combined Systems	Germination Percentage	0.66	0.36	0.37	0.53
	Days to Germination	0.54	0.51	0.62	0.75
	Vigor	0.78	0.78	0.82	0.83
Organic	Germination Percentage	0.45	0.36	0.66	0.71
	Days to Germination	0.30	0.33	0.45	0.63
	Vigor	0.47	0.57	0.61	0.72
Conventional	Germination Percentage	0.52	0.14	0.12	0.18
	Days to Germination	0.33	0.35	0.26	0.80
	Vigor	0.58	0.72	0.72	0.73

### 3.2 Changes in root disease and morphology across breeding history, parentage, and seed color

ANOVA indicated that several of the examined factors – seed color, breeding history, cross, production system, and the production system × breeding history interaction – had a significant influence on root morphology and disease incidence (α < 0.05), as shown in **Table 5** and **Supplementary Table 3**. Seed color, as a main effect, was significantly associated with taproot and basal root diameter. Breeding history had a significant effect on basal root diameter, branching density, and disease. Parental cross significantly influenced branching density and disease. Production system and the production system × breeding history interaction had a significant effect on all traits except taproot diameter. Parental cross, production system, and breeding history had the largest effects on disease incidence, respectively.

**Table 5:** Estimated marginal means (EMMs) with standard error for root disease and branching density across breeding history and parentage contrast factors.

Production System		Root Disease (0-9 Scale)				Branching Density (0-5 Scale)			
		Breeding History		Cross		Breeding History		Cross	
		Organic	Conventional	ORBV	HYPR	Organic	Conventional	ORBV	HYPR
All Families	Combined Systems	1.42 ± 0.05^a^	1.60 ± 0.05^b^	1.39 ± 0.05^a^	1.64 ± 0.05^b^	4.24 ± 0.08^a^	3.86 ± 0.08^b^	3.72 ± 0.08^a^	4.38 ± 0.08^b^
	Organic	1.57 ± 0.07^a^	1.78 ± 0.08^b^	1.51 ± 0.08^a^	1.84 ± 0.07^b^	4.10 ± 0.11^a^	3.92 ± 0.12^a^	3.88 ± 0.12^a^	4.14 ± 0.11^a^
	Conventional	1.29 ± 0.07^a^	1.44 ± 0.07^b^	1.27 ± 0.07^a^	1.46 ± 0.06^b^	4.35 ± 0.12^a^	3.74 ± 0.12^b^	3.54 ± 0.13^a^	4.55 ± 0.12^b^
White-Seeded Families	Combined Systems	1.32 ± 0.09^a^	1.79 ± 0.11^b^	1.48 ± 0.12^a^	1.63 ± 0.09^a^	4.45 ± 0.16^a^	3.38 ± 0.19^b^	3.40 ± 0.21^a^	4.43 ± 0.15^b^
	Organic	1.52 ± 0.17^a^	2.11 ± 0.21^b^	1.68 ± 0.22^a^	1.95 ± 0.16^a^	4.04 ± 0.25^a^	3.30 ± 0.31^b^	3.53 ± 0.31^a^	3.81 ± 0.25^a^
	Conventional	1.11 ± 0.09^a^	1.50 ± 0.11^b^	1.28 ± 0.12^a^	1.33 ± 0.08^a^	4.83 ± 0.22^a^	3.49 ± 0.25^b^	3.31 ± 0.28^a^	5.02 ± 0.20^b^
Pigmented Seed Families	Combined Systems	1.46 ± 0.06^a^	1.57 ± 0.06^a^	1.39 ± 0.06^a^	1.64 ± 0.06^b^	4.17 ± 0.07^a^	3.93 ± 0.07^b^	3.75 ± 0.07^a^	4.34 ± 0.08^b^
	Organic	1.51 ± 0.07^a^	1.63 ± 0.07^a^	1.42 ± 0.07^a^	1.73 ± 0.07^b^	4.32 ± 0.11^a^	4.29 ± 0.11^a^	4.16 ± 0.10^a^	4.45 ± 0.11^a^
	Conventional	1.40 ± 0.08^a^	1.50 ± 0.08^a^	1.35 ± 0.08^a^	1.55 ± 0.08^b^	4.01 ± 0.14^a^	3.57 ± 0.14^b^	3.35 ± 0.14^a^	4.23 ± 0.15^b^

Means followed by different superscript letters indicate significant pairwise differences between factor levels, based on Tukey’s HSD test (α < 0.05).

**Figure 2:**
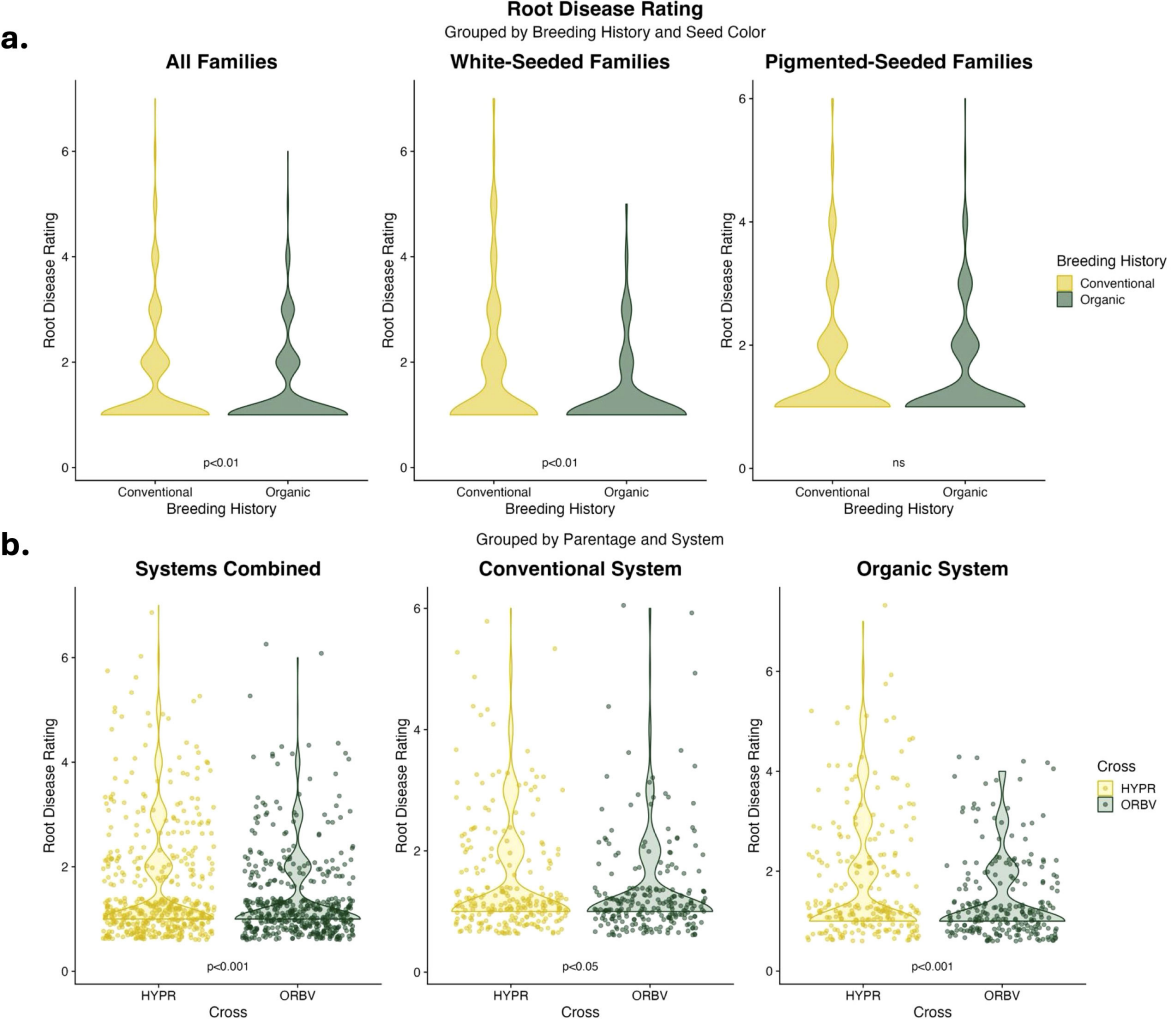
**(a)** Violin plots of root disease data collected on F_8_ families in 2021. Data are categorized by seed color and breeding histories, and were analyzed across populations and production systems. Significance values are derived from Tukey HSD tests. **(b)** Violin plots of root disease data collected on F_8_ families in 2021. Data are categorized by production system and parentage, and were analyzed across breeding history, populations and seed color. Significance values are derived from Tukey HSD tests.

**Figure 3:**
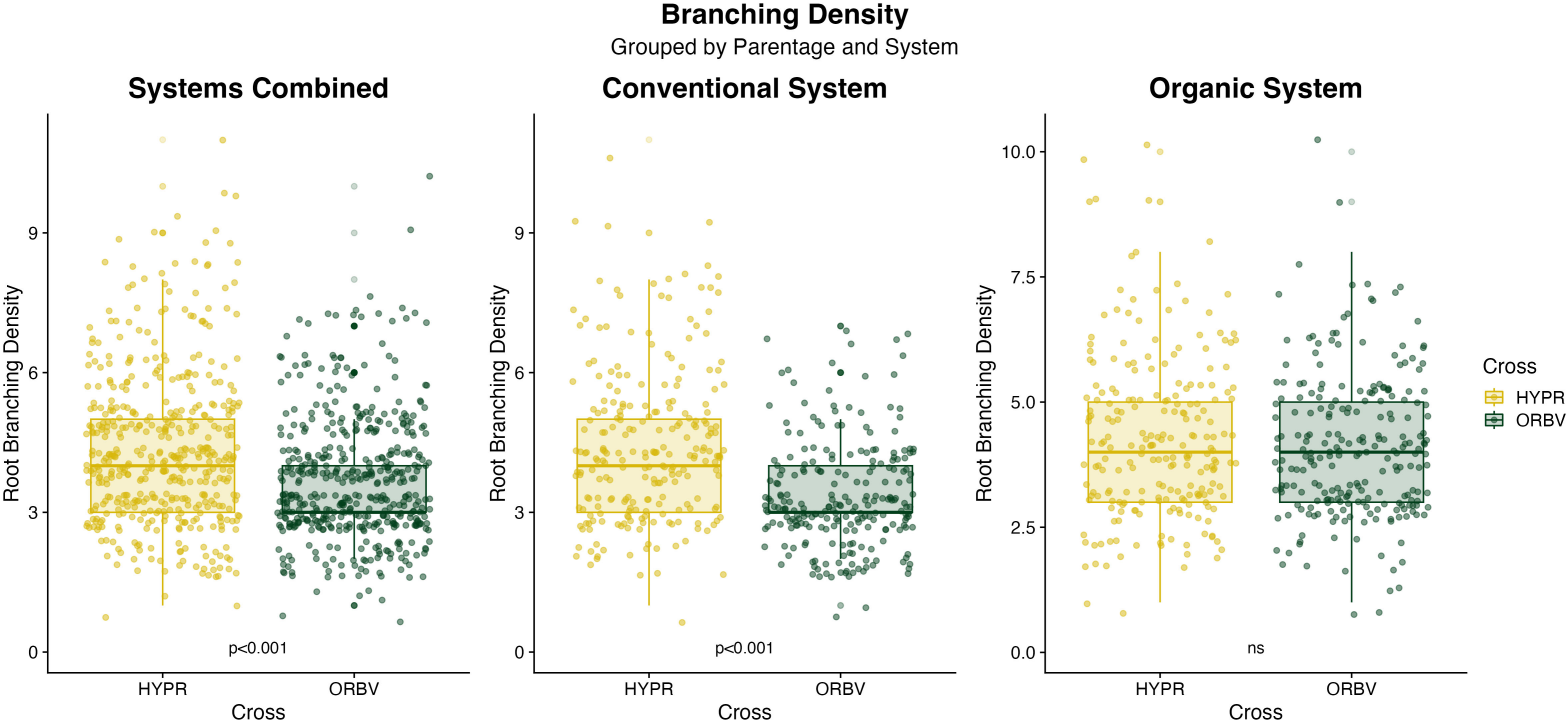
Boxplots of branching density data collected on F_8_ families in 2021. Data are categorized by production system and parentage, and were analyzed across breeding history, populations and seed color. Significance values are derived from Tukey HSD tests.

Root disease was lower among organically-bred families, as shown in **Figure 2a**. This reduction was significant (α < 0.05) across all families. When the families were subset for white and pigmented seed color, the organically-bred families showed a significant reduction in root disease amongst the white-seeded families, but disease ratings across the pigmented seed families did not differ significantly between breeding histories. There were also significant decreases (α < 0.05) in root disease among the ORBV families, shown in **Figure 2b**. This decrease was significant across production systems, as well as within each production system. The ORBV pigmented seed families primarily contributed to the significant disease reduction, and we found no significant reduction of disease in the ORBV white-seeded families. EMMs for root disease data are shown in **Table 5**. In addition to disease reduction, parentage significantly affected root branching density, as shown in **Figure 3**. Families derived from the HYPR cross had significantly higher (α < 0.05) branching density than ORBV families across production systems and within the conventional system; however, this relationship was not broadly identified in the organic production system. EMMs for branching density are shown in **Table 5**.

Narrow-sense heritability, calculated for traits observed in a single environment, ranged from low to moderate heritability for root-related traits (**Table 6**). Heritability of branching density ranged from 0.02 to 0.26 across production systems. For disease rating, the heritability ranged from 0 to 0.25 across production systems. Heritability of root diameter ranged from 0 to 0.46.

**Table 6:** Narrow-sense heritability of traits collected in one year (2021) and environments, for each population.

Production System	Trait	Narrow-Sense Heritability			
		ORBV-O	ORBV-C	HYPR-O	HYPR-C
Organic	Basal Root 1 Diameter	0.08	0.12	0.44	0.28
Conventional	Basal Root 1 Diameter	0.00	0.06	0.17	0.04
Organic	Basal Root 2 Diameter	0.16	0.06	0.32	0.46
Conventional	Basal Root 2 Diameter	0.13	0.01	0.07	0.00
Organic	Taproot Diameter	0.13	0.26	0.37	0.26
Conventional	Taproot Diameter	0.00	0.00	0.21	0.04
Organic	Branching Density	0.16	0.12	0.02	0.16
Conventional	Branching Density	0.21	0.02	0.25	0.26
Organic	Disease Rating	0.25	0.08	0.05	0.00
Conventional	Disease Rating	0.00	0.00	0.00	0.06
Organic	100 Seed Weight	NA	NA	NA	NA
Conventional	100 Seed Weight	0.84	0.77	0.72	0.75
Organic	Days to 50% Flower	0.74	NA	0.73	NA
Conventional	Days to 50% Flower	NA	0.68	NA	0.74
Organic	Seed Weight/Plant (grams)	0.40	NA	0.21	NA
Conventional	Seed Weight/Plant (grams)	NA	0.48	NA	0.44

### 3.3 Multiple correlations analysis

Spearman’s rank correlations, shown in **Supplementary Table 6**, indicated a moderate and significant positive correlation between germination percentage and seed pigmentation across all three years. In all years, this relationship was stronger within the conventional populations than in the organic populations. In 2018, seed pigmentation was slightly negatively correlated with individual plant yield (i.e., seed weight per plant) in the organic populations, but it had a slightly positive correlation in the conventional populations. In 2020, seed pigmentation was more strongly correlated with 100-seed weight in the organic populations, and we found a higher correlation between days to germination and 100-seed weight in the organic populations. Days to germination and germination percentage had strong negative correlations in both breeding histories in 2020. Seed pigmentation was negatively correlated with days to germination in the conventional populations in 2021, but we found no significant correlation between these traits in the organic populations.

We identified positive correlations between taproot and basal root diameters for both organic and conventional populations in 2021. Branching density had a slight positive correlation with days to germination in the organic populations and a slight negative correlation with days to germination in the conventional populations. Negative correlations were also identified between branching density and germination percentage in the organic populations only.

Next, we explored correlations between the same traits but within the pigmented and white-seeded families of each population (**Supplementary Table 7**). In the 2018 white-seeded families, seed weight per plant was positively correlated with germination percentage in the conventional populations but negatively correlated in the organic populations. In 2020, the white-seeded families had stronger negative correlations between germination percentage and days to germination than the pigmented seed families. For the 2021 data, we identified positive correlations between germination percentage and days to germination, but only among the pigmented seed families. Differences in these correlations between the conventional and organic production systems were also explored, as shown in **Supplementary Table 8**. Trends were similar to those shown in **Supplementary Table 7**. In 2021, there was notably a stronger negative correlation between germination percentage and days to germination among the white-seeded families grown in the organic system than in the conventional system.

All correlations discussed and shown in the **supplementary materials** were significant (α<0.05).

### 3.4 Genotype × environment interactions vary according to parentage

In order to evaluate G × E interactions, we utilized a series of mixed models to estimate marginal means (EMMs) for each snap bean population, followed by Type-III tests of the fixed effects on the resulting EMMs. Our results (shown in **Supplementary Table 9**) suggested that variation existed between population parentage, so we supplemented the analysis by running the models with all data combined and separately with subsets of ORBV and HYPR parentage. Results for the G × E interaction factor are shown in **Table 7**, and results for all model factors are shown in **Supplementary Table 10**.

**Table 7:** Genotype-by-environment effect terms for all snap bean traits evaluated in 2020 and 2021.

Trait	Parental Cross	F stat	d.f.
Germination Percentage			
	Combined	14.873***	3
	ORBV	ns	1
	HYPR	ns	1
Days to Germination			
	Combined	18.121***	3
	ORBV	49.819***	1
	HYPR	5.240*	1
Vigor			
	Combined	14.620***	3
	ORBV	11.653***	1
	HYPR	28.563***	1
Taproot Diameter			
	Combined	4.569**	3
	ORBV	6.126*	1
	HYPR	4.753*	1
Basal Root 1 Diameter			
	Combined	5.912***	3
	ORBV	ns	1
	HYPR	7.599**	1
Basal Root 2 Diameter			
	Combined	4.728**	3
	ORBV	ns	1
	HYPR	7.786**	1
Branching Density			
	Combined	14.642***	3
	ORBV	ns	1
	HYPR	16.674***	1
Disease Rating			
	Combined	6.178***	3
	ORBV	11.213***	1
	HYPR	4.997*	1

Factor effects are shown for the full dataset, as well as for subsets associated with each of the two parentages (ORBV and HYPR). *ns*: *p* > 0.05, *: *p* ≤ 0.05, **: *p* ≤ 0.01, ***: p≤ 0.001.

**Figure 4:**
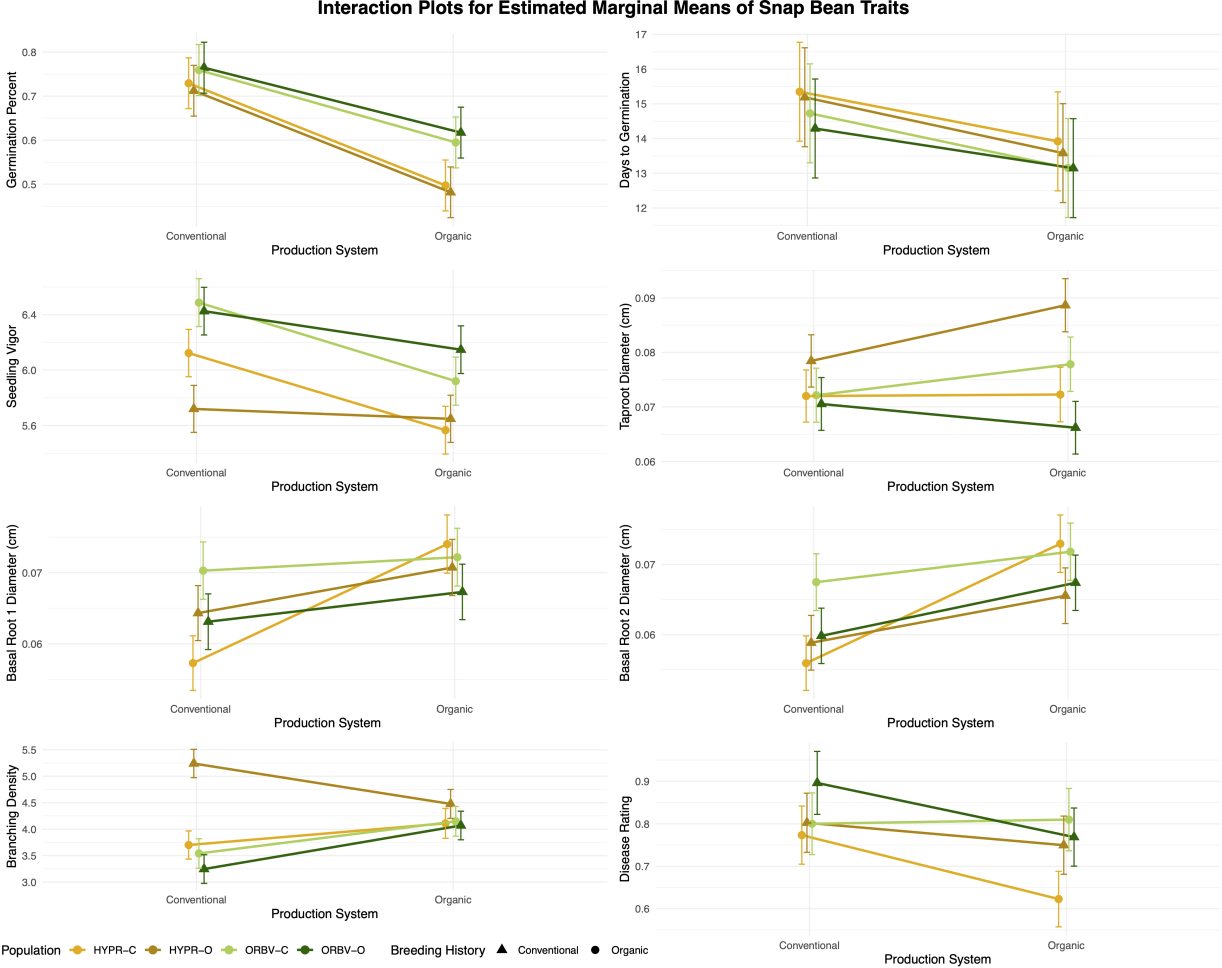
Genotype × environment interaction plots for early-season and root traits in snap bean populations. Estimated marginal means (EMMs) were generated for each population within each production system, using data collected from F_7_ and F_8_ families in 2020 and 2021.

In the models generated for G × E interactions, the production system factor was significant across all evaluated traits and parentage groupings, with the exception of taproot and basal root 1 diameters in the ORBV populations. The population factor was also significant for many traits, but not across all parentage groupings. Significant G × E interactions, visualized in **Figure 4**, were identified for many traits, with variation across parentage groups.

For germination percentage, the interaction term was only significant when parentage groups were combined, indicating that there was no genotype × environment interaction between related populations of opposite breeding histories. In the other early-season performance traits, days to germination and vigor, significant interactions were identified in both populations for both traits. For days to germination, the population trends were divergent. In the HYPR populations, days to germination was longer in the HYPR-C population than in HYPR-O in both production systems, with the difference between populations increasing marginally in the organic system. In the ORBV populations, ORBV-C took longer to germinate than ORBV-O in the conventional system, but the population means were nearly identical in the organic system. For vigor, the population trends revealed a crossover interaction, in which the organically-bred populations had lower vigor than their conventional counterparts in the conventional system but higher yield in the organic system. These results demonstrate that populations vary in their performance according to the production system, and that useful variation may be captured to improve the early-season performance of plants in a specific growing environment.

Among the root traits, G × E interactions were less straightforward. For example, in the root diameter traits, the ORBV populations showed consistent trends of higher diameter among the conventional populations and in the organic production system. For the same traits in the HYPR populations, HYPR-O had thicker taproots in both systems but a crossover interaction was observed with HYPR-C for the basal root diameters. Branching density was similar among HYPR-C, ORBV-O, and ORBV-C, with lower density in the conventional system. HYPR-O deviated from this pattern, exhibiting much higher branching density in the conventional system. For root disease, the parentage group patterns deviated. In the HYPR populations, HYPR-O had higher disease in both systems, with the difference increasing in the organic system. In the ORBV populations a crossover effect was observed where ORBV-O had higher disease than ORBV-C in the conventional system, but lower disease in the organic system. Although more variable, the results for the root traits still clearly demonstrate population-specific responses to differing environments.

### 3.5 QTL mapping

Using the BARCBean12K BeadChip SNP data, we constructed linkage maps for all four populations. **Table 8** details map information for each of the sets of linkage maps. The HYPR linkage maps are included in **Supplementary Figures 1** and **2**. The ORBV linkage maps are included in **Supplementary Figures 3** and **4**.

**Table 8:** Numbers of markers pre-and post-JoinMap, individuals used in map after removing heterozygous and identical individuals, and overall cM of each map.

Mapping Population^x^	Generation	Year	Polymorphic Markers (no.)	Markers in Final Map (no.)	Population size in Final Map (no.)	Coverage (cM^y^)
ORBV-O	F_8_	2022	3086	295	79	991.36
ORBV-C	F_8_	2022	3086	398	86	929.74
HYPR-O	F_7_	2020	2084	285	92	867.79
HYPR-C	F_7_	2020	2084	357	88	813.26

x Mapping populations are described in detail in **Table 1**.

y Based on 1,200 cM average genetic map length (Gepts, 2001).

After initial filtering, we identified 3086 polymorphic markers for the ORBV populations and 2084 polymorphic markets for the HYPR populations. After additional SNP evaluation, 295 markers were retained in the ORBV-O maps, 398 markers were retained in the ORBV-C maps, 285 markers were retained in the HYPR-O maps, and 357 markers were retained in the HYPR-C maps. This represented coverage of 991.36, 929.74, 867.79, and 813.26 cM, respectively (**Table 8**). The HYPR populations generally had lower coverage compared to the ORBV populations. While the organic breeding history maps generally had fewer markers, they had higher coverage compared to the conventional breeding history maps. QTL found with the associated maps for each population are discussed below.

A total of 78 QTL were found for the traits used in this study, and these QTL were dispersed throughout all eleven linkage groups, with the exception of LG 10 (**Supplementary Table 11**). Maximum LOD scores ranged from 2.74 for a QTL linked to days to germination (DTG) on LG 6 to 12.85 for a QTL linked to germination percentage on LG 7. The phenotypic variation explained (R^2^) by the QTL ranged from 0.13 for DTG on LG 6, to 0.5 for a germination percentage QTL and for two vigor QTL on LG 7.

#### 3.5.1 Days to germination

We identified several genetic regions linked to days to germination (**Supplementary Table 11**). Both ORBV populations showed a significant QTL on LG 7 for the 2020 growing season in the organic system (also in the conventional system for ORBV-O). The ORBV-O population also had significant QTL on LG 2_1 in the conventional system in 2021, LG 4 in the conventional system in 2020, and LG 8 in both systems in 2021.

As in the ORBV populations, there was a significant QTL for both populations on LG 7. There was also a significant QTL for both populations on LG 6-1. This location was significant for the HYPR-O population in the organic system in 2020. This locus was more significant in the HYPR-C population in both the 2020 organic and conventional systems, and in the organic system in 2021. There was also a significant QTL on LG 4 for the HYPR-C population in the organic system in 2021.

#### 3.5.2 Germination percentage

The ORBV-O population had a significant QTL on LG 4 in the 2020 organic and conventional systems, as well as one on LG 7 in the organic system in 2021 (**Supplementary Table 11**). The ORBV-C population had significant QTL on LG 7 in 2018 and in the organic system in 2021. The ORBV-C population also had a significant QTL on LG 8–1 in the conventional system in 2020.

For the HYPR-O population, we identified significant QTL on LG 7 in the organic system in 2018 and 2021, and QTL in different regions for the conventional system, LG-2 in 2020 and LG 4 in 2021. For the HYPR-C population, there was a significant QTL on LG 7 in the organic system in 2020 and both systems in 2021. The HYPR-C population also had a significant, stable QTL on LG 6–1 in 2018 and in both systems in 2020 and 2021. This region was not significant in the HYPR-O population.

#### 3.5.3 Days to flowering

We identified a significant QTL related to days to flowering for the ORBV-O population on LG 3, and significant regions for the ORBV-C population on LG 1–2 and LG 8-1 (**Supplementary Table 11**).

Both HYPR populations had significant QTL for days to flowering on LG 4 and 5, both of which appear to overlap. The LODs suggest a stronger significance in the organic, especially on LG 5 (LOD 7.66). The conventional population had an additional significant QTL on LG 11.

#### 3.5.4 Root traits

We did not identify any significant QTL for root traits in the ORBV populations.

Both HYPR populations had significant QTL for basal root diameter on LG7, and the HYPR-C population also had a significant QTL for taproot diameter at the same location (**Supplementary Table 11**). The HYPR-C population had a significant QTL for root branching density on LG 1. The only significant QTL for disease were in the HYPR-C population, one on LG 2 in the organic system in 2021 and one on LG 3 in the conventional system in 2021.

#### 3.5.5 Vigor

We found a significant QTL for vigor on LG 7 in the ORBV-O population in the 2020 conventional system and both systems in 2021 (**Supplementary Table 11**). The ORBV-O population had additional significant QTL in the conventional system, on LG 4 in 2020 and LG 6 in 2021. In the ORBV-C population, we identified significant QTL for vigor in the ORBV-C population in both systems in 2020 and 2021. ORBV-C had an additional significant locus on LG 9 in the 2020 conventional system.

We identified one significant QTL for vigor in the HYPR-O population on LG 7 in the organic system in 2021. The HYPR-C population had a significant QTL on LG 7 in both systems in 2020 and 2021. We found an additional significant QTL in the HYPR-C population in the 2020 organic system on LG 4 and LG 6-1.

#### 3.5.6 Seed traits

Only the ORBV-C population had significant QTL for 100-seed weight (**Supplementary Table 11**). The loci, found on linkage groups 2, 5, and 7, were stable in 2018 and 2020. Both ORBV populations had a significant QTL on LG 7 for seed color.

As in the ORBV populations, only the HYPR conventional population had significant QTL for 100-seed weight. We found a significant QTL on LG 3 in 2020, and a more stable QTL on LG 7 in 2018 and 2020. We identified a significant QTL for seed color in the HYPR populations on LG 7. The HYPR-O populations had a significant QTL on LG 7 for seed weight per plant, whereas for the HYPR-C population we identified a significant QTL for this trait on LG 1 and LG 6-1.

#### 3.5.7 Stable QTL

Of the QTL listed above, a number of these regions were stable over two or more years (**Supplementary Table 11**).

We identified stable QTL in the HYPR populations for days to germination on LG 6, in the 16.8-24.2 Mbp region. This region was significant in HYPR-O and HYPR-C in the 2020 organic system, and again in the 2021 organic system for the HYPR-C populations. We also found a QTL related to germination percentage in the HYPR-C population that overlapped this region (3.3-24.4 Mbp) across systems in 2018, 2020, and 2021.

The ORBV and HYPR populations had significant QTL in at least two years on LG 7 for germination percentage, vigor, and 100-seed weight. We identified stable QTL for germination percentage in the ORBV populations on LG 7, in the 8.7-30.4 Mbp region. These QTL were significant for the ORBV-C population in 2018 and 2020, as well as for the ORBV-O population in 2021. In the HYPR populations, we identified several QTL for germination percentage over multiple years and systems in the 7.5-36.7 Mbp region of LG 7. For seedling vigor in the ORBV, we found several significant QTL on LG 7, between 1.6 Mbp and 30.7 Mbp. In the HYPR populations, we identified similar QTL on linkage group 7, including a QTL for the HYPR-O population in the 2021 organic system in the 8.4-36.7 Mbp region and a stable QTL for the HYPR-C population in the 7.5-35.2 Mbp region in both systems in 2020 and 2021. For 100-seed weight, we found significant QTL in the conventional populations and in the conventional system. The ORBV-C population had three stable QTL related to 100-seed weight on three different linkage groups: 2 (24.9-34.1 Mbp), 5 (9.3-38.2 Mbp), and 7 (3.3-8.8 Mbp). We identified a significant QTL for 100-seed weight in the HYPR-C populations on LG 7 in the 25.9-35.2 Mbp region. There were many QTL in all populations linked to days to germination on LG 7 in the 1.6-38.2 Mbp region.

### 3.6 Segregation distortion

**Figure 5:**
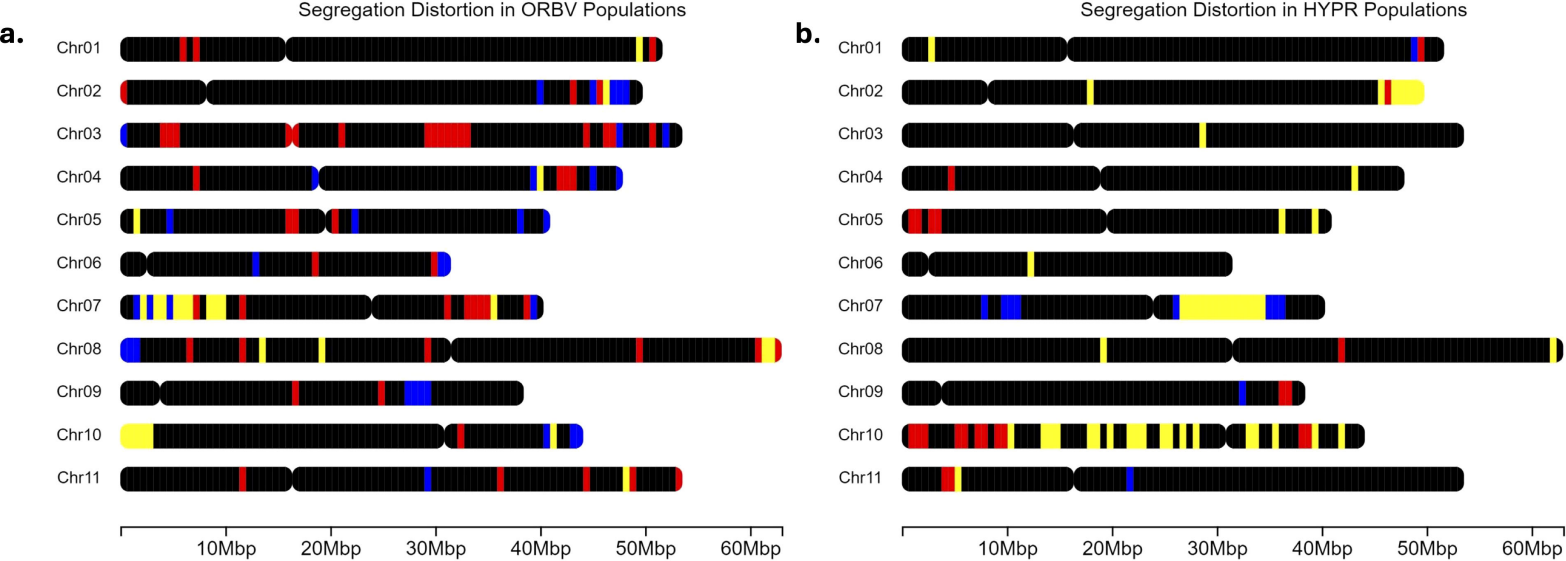
**(a)** Segregation distortion throughout the genome in the OR5630 × Black Valentine (ORBV) populations. Blue regions correspond to regions uniquely distorted in the organic populations, red to regions in conventional populations, and yellow indicates shared regions of distortion. Black regions are either monomorphic or follow expected Mendelian ratios. Chromosome length is based on all SNPs from the BARCBean12K BeadChip. **(b)** Segregation distortion throughout the genome in the Hystyle × Provider (HYPR) populations. Blue regions correspond to regions uniquely distorted in the organic populations, red to regions in conventional populations, and yellow indicates shared regions of distortion. Black regions are either monomorphic or follow expected Mendelian ratios. Chromosome length is based on all SNPs from the BARCBean12K BeadChip.

Distorted SNPs found in each population are reported in **Supplementary Table 12** and plotted in **Figures 5A** (ORBV) and 5b (HYPR). A summary of the SNPs found in each population and the chromosomes on which they occur is presented in **Supplementary Table 13**.

For the ORBV populations, there were 3,086 SNPs after filtering for monomorphic markers and missing data. Among these markers, we found that 277 were distorted SNPs in the organic population and 241 in the conventional (**Figure 5a**). We determined that 74 of these markers were distorted in both populations (‘shared’ on **Figure 5a**), and these markers occurred on every linkage group except LG 3, 6, and 9. Both populations had unique SNPs, or regions of distorted SNPs, on every linkage group, except for the organic population on LG 1. The ORBV-O population had significantly more distorted markers on LGs 2, 9, and 11, while the ORBV-C population had more distortion on LGs 3, 4, and 7. When comparing the ORBV populations, ORBV-O had more distorted markers on LG 2, LG 5, LG 6, LG 9, LG 10, and LG 11. The ORBV-C population had more distorted SNPs than ORBV-O on LG 1, LG 3, LG 4, and LG 7.

For the HYPR populations, we found 2,084 SNPs after filtering. Of these, 234 markers were distorted in the organic population, 309 were distorted in the conventional population, and 196 were distorted in both populations (**Figure 5b**). For markers distorted in both populations, we found large distortion regions on LGs 2, 7, and 10. There was a strong skew towards the maternal allele for the regions on LG 2 in both populations, with a higher frequency in the organic population. The shared region on LG 10 was also skewed towards the maternal allele in both populations, to a lesser degree than those on LG 2. Both populations skewed towards the paternal allele in the regions on LG 7. For the HYPR populations, HYPR-O had more distorted SNPs on LG 1 and LG 7, and HYPR-C had more distortion on LG 2, LG 4, LG 5, LG 8, LG 10, and LG 11.

## 4 Discussion

Organic production environments expose crops to distinct stressors that are typically minimized through chemical interventions in conventional systems. Breeding crops that are resilient to these stressors is a predominant goal within organic agriculture; however, the traits associated with resilience in organic systems are difficult to identify and select for due to the high variation in organic growing systems and the complex, quantitative inheritance of these traits. Our work sought to identify specific traits in snap beans that are beneficial for growth in organic production environments, along with finding regions to develop genetic markers that are associated with these traits. To achieve this, we developed four populations of snap beans that allowed us to make comparisons across parentage and breeding history. We anticipated that the organic populations would outperform the conventional populations within organic production systems, and our findings did point to significant crossover interactions in the organic and conventional populations. Furthermore, the narrow-sense heritability values for many traits indicated similar, and in some cases, higher heritability in the organic environments compared to the conventional environments. These values show that breeding within organic environments is a viable and potentially preferable approach to developing varieties that are well-suited to organic production systems.

To explore population variation for germination-related traits, we enacted high abiotic (i.e., cold and wet soils) and biotic (i.e., pathogenic fungi) pressure on seedlings through extraordinarily early planting dates. As anticipated, the production system significantly influenced germination-related traits. In general, total germination was lower within the organic production environments; yet, an exploration of the role of seed color indicated that this gap between production systems was largely due to the white-seeded cultivars (**Figure 1a**). Our planting dates corresponded to suboptimal germination temperatures (i.e., below 13°C), under which common bean seed imbibes moisture more slowly and thus takes longer to germinate (Walls and Stang, 1985). The reduction in germination seen under cold soil temperatures is exacerbated in white-seeded beans, as white seed is often correlated with a higher rate of imbibitional cracking and injury (Cirak and Myers, 2021). Although our white-seeded families had lower germination, those from an organic breeding history had consistently higher germination than those from a conventional breeding history. In addition to the variation in germination percentage, we identified significantly faster germination among the white-seeded families of organic breeding history compared to their conventional counterparts (**Figure 1b**). Plasticity in germination as a response to environmental conditions may explain this increase in speed along with increases in soil temperature that may have resulted from compost application, as others have reported (Deguchi et al., 2009; Donohue et al., 2005). Furthermore, a growing body of evidence suggests the bean seed microbiome may provide plan-growth-promoting properties to the germinating seeds, allowing for faster germination in the presence of pathogenic microbes (Martins et al., 2018; Mokrani et al., 2019). Given the increased abiotic and biotic pressures that are inherent to the early growing season, this acceleration of germination can be interpreted as an advantageous escape strategy. Accelerated imbibition and germination of white-seeded beans are understood to be flaws, which put the germinating seeds at higher risk of chilling injury or pathogen attack; however, our findings suggest that this characterization of white-seeded bean performance is incomplete and additional genetic factors may have a role in early-season resilience. Vidak et al. (2022) found similar results suggesting that while seed coat color has a significant role in bean germination, it does not explain all of the variation in germination and seedling emergence. Thus, we understand that there may be alternative routes to improving germination in white-seeded beans without altering seed color. This is singularly important for the organic snap bean processing market, where white seeds are essential to the product quality of processed beans but can simultaneously contribute to the organic yield gap through reduced germination in the field.

Similar to results for early-season performance, we found that the production system significantly influenced root morphology and disease. Other factors, including breeding history and cross, also significantly influenced root and disease traits. Many of these root traits were minimally explained by study factors, except for root branching density and disease development. Root disease was significantly lower within organically-bred families, compared to conventional counterparts, and this variation was largely explained by differences among the white-seeded families (**Figure 2a**). Faster germination has been suggested as an effective means through which *P. vulgaris* seedlings may escape and outgrow soilborne pathogenic pressures, particularly those that cause damping-off (i.e., *Pythium*, *Rhizoctonia*, etc.) (Li et al., 2015; Peña et al., 2013). Thus, the changes observed in germination speed among white-seeded families from an organic breeding history may partially explain these specific reductions in root disease. Comparing across parentage, the ORBV families had significantly lower root disease compared to the HYPR families (**Figure 2b**). This difference was not surprising, given the disease resistance package of OR5630 and previous research into the root rot susceptibility of the four parental lines, which determined that Black Valentine has high resistance to the root rot complex prevalent in western Oregon (Huster et al., 2021). These findings deviate from other work with the four RIL populations, which identified a trend towards higher pathogen abundance in the rhizosphere soil of ORBV populations; however, these results were collected from a separate field site and pathogen abundance was determined through a metabarcoding approach that was independent of root disease phenotyping (Park et al., 2023). Branching density was significantly higher among the HYPR families, indicative of a denser root system (**Figure 3**). Organically-bred families also had significantly higher branching density, compared to the conventionally-bred families. Other work indicates that higher lateral root branching density significantly increases the ability of roots to cope with stressors, particularly in phosphorus-limited soils, suggesting that increased branching density may be an advantageous root system adaptation for nutrient-limited organic environments (Postma and Lynch, 2011).

Positive correlations between seed pigmentation and germination percentage were identified across all years and populations, although these correlations were higher among conventionally-bred populations. Seed pigmentation positively correlated with seed weight, a trend that was stronger among organically-bred populations. Seed weight, however, was positively correlated with days to germination and negatively correlated with germination percentage, suggesting that the larger seeded beans were slower to emerge or failed to emerge altogether. These results correspond to our findings that white-seeded organic families germinate faster, as well as other studies that have identified larger, pigmented seeds to be slower to germinate and thus depleted in field emergence (De Ron et al., 2016; Vidak et al., 2022).

Analysis of G × E relationships across traits (**Figure 4**) confirmed the importance of breeding within a target environment. Days to germination showed significant G × E interactions, with population trends differing by parentage. In HYPR populations, HYPR-C germinated later than HYPR-O in both systems, with a slightly greater difference in the organic system. In ORBV populations, ORBV-C germinated later than ORBV-O in the conventional system, but both populations had similar germination times in the organic system. No interactions were observed for germination percentage, but germination was consistently lower in the organic systems. This trend is likely explained, in part, by the presence or absence of the fungicide seed treatment, which is intended to reduce seedling mortality due to biotic pressures. Notably, we identified a crossover interaction for seedling vigor, with organically-bred populations performing better in organic systems and conventionally-bred populations performing better in conventional systems. This interaction was stable across both parentage groups (ORBV and HYPR), suggesting that breeding within an organic system can enhance seedling vigor of progeny grown under organic conditions. We identified a crossover interaction in ORBV populations for root rot disease severity (higher in ORBV-O in the conventional system but lower in the organic system). This interaction was not observed in the HYPR populations, where HYPR-O had consistently higher disease severity. The interaction in the ORBV populations suggests organically bred families may have developed resistance mechanisms specific to organic environments, whereas the HYPR populations’ inconsistent response raises questions about the stability of this interaction. Previous research into the root rot susceptibility of the Common Bean Coordinated Agricultural Project (BeanCAP) Snap Bean Diversity Panel classified both OR5630 and Black Valentine as highly resistant to the western Oregon root rot complex, which may partially explain the variation observed in G × E interactions (Huster et al., 2021). Other root traits showed less straightforward patterns, suggesting complex interactions between genotype and soil conditions. These findings are not unexpected, as root traits frequently show high plasticity in field environments (Strock et al., 2019; Vieira et al., 2008). This plasticity may itself be advantageous, as suggested by Burridge et al. (2016) and Zhu et al. (2010), but the trends observed in this study do not point to any consistent genotypic variation. Although our sampling protocol sought to characterize true variation in field-grown populations, the use of more controlled assays, such as *in vitro* germination tests, may result in clearer population trends. The significance of the production system across nearly all traits confirms that environmental factors tied to organic or conventional management can strongly influence phenotypes. These results emphasize the importance of breeding within organic environments to bolster adaptation, particularly for traits showing strong environmental dependencies (i.e., vigor, disease, root architecture). The observed crossover interactions indicate potential trade-offs between conventional and organic selection, underscoring the need for breeders to strategically prioritize traits based on the specific demands of each production system.

Heritability analysis in early-season and root system traits indicated consistent heritability values across production systems. For certain traits, namely germination percentage, seedling vigor, root diameters, and disease rating, the heritability was higher in the organic production system than in the conventional production system. These findings, in conjunction with improved performance of organically-bred populations within the organic environment, support the theory of optimizing the correlation of genotype performance in specific test environments and in the target population of environments within breeding programs.

The genetic linkage maps generated for this work had between 285 and 398 markers, and a coverage ranging from 813.26 to 991.36 cM, which correspond to 78 and 95 percent coverage, respectively, of the Stampede x Red Hawk map (Song et al., 2015). The final marker number was an improvement over initial maps made for the populations, described by King (2019); however, the final maps had fewer markers than those generated for other mapping populations that utilized the BARCBean6K BeadChip or the BARCBean12K BeadChip (Bassett et al., 2021; Heilig et al., 2017). We attribute this primarily to the relatively small population sizes, approximately 90 families within each population. Furthermore, we anticipate that the low marker number in the HYPR maps was a result of the parental background, as both parents originate from the Andean gene pool (Wallace et al., 2018). One of the parents of the ORBV populations, ‘OR5630’, is derived from both Andean and Middle American backgrounds. The other parent, ‘Black Valentine’, originates from the Andean gene pool. The ORBV populations had greater genetic diversity amongst the loci covered, likely due to these differences in the gene pools of the parents (Wallace et al., 2018). Quantitative trait loci (QTL) analysis was conducted to identify chromosomal regions linked to important traits for *Phaseolus vulgaris*, and many of these loci aligned with regions found in other studies (**Supplementary Table 11**). QTL with higher values of phenotypic variation (R^2^) suggest the effect of additive genes in the control of traits. Many QTL were near or overlapping regions noted in the Arriagada et al. (2022) and Izquierdo et al. (2023) meta-QTL analyses for important bean yield traits, including 100-seed weight on LGs 2, 3, 5, and 7; days to flowering on LGs 1, 3, 4, 5, and 11; and seed yield on LGs 1, 6, and 7. Additionally, the QTL we found for 100-seed weight on Pv07 is consistent with several other studies (González et al., 2017; Koinange et al., 1996; Pérez-Vega et al., 2010). A QTL we identified for root disease on LG 3 contained a significant SNP (12,661,037 bp) that Huster et al. (2021) identified for the same trait in a genome-wide association study (GWAS). Hagerty et al. (2015) found a QTL linked to *Fusarium solani* resistance that was also located on LG 3. We identified several QTL linked to root diameter on LG 7, similar to López-Marín et al. (2009).

There were several notable trends for the quantitative trait loci found for these populations. A number of the identified QTL were stable over two or more years and in multiple growing systems. These regions included the following: days to germination on LG 6_1; germination percentage on LG 6_1, and LG 7; vigor on LG 7; 100-seed weight on LG 2, LG 5, and LG 7. The ORBV and HYPR populations had significant QTL in at least two years on LG 7 for germination percentage, vigor, and 100-seed weight. One-hundred seed weight on LG 7 is consistent with González et al. (2017); Koinange et al. (1996), and Pérez-Vega et al. (2010). Variation in seed related traits is highly influenced by seed color and important seed proteins (Johnson et al., 1996). Both the *P* locus, which has been linked to seed pigmentation in beans, and phaseolin, one of the most important seed proteins in beans, have been found on LG 7 (Johnson et al., 1996; Koinange et al., 1996; McClean et al., 2018; Moghaddam et al., 2014). One of the two stable regions found for 100 seed weight on LG 7, with a peak in the 32.3 Mb region, was within a meta-QTL identified by Izquierdo et al. (2023) that contained a serine/threonine-protein kinase (STK) gene (*Phvul.007G174900*). STK genes have been linked to flowering and seed weight in rice, and grain yield in maize (Deng et al., 2017; Hu et al., 2012; Jia et al., 2020). In the same study, this same region on LG 6_1 corresponded with a meta-QTL noted to contain a receptor associated kinase (RAK) (*Phvul.006G020700*) that was associated with seed yield, seed weight, and days to physiological maturity. These two stable regions on LG 6_1 and LG 7 also overlap with meta-QTL in Izquierdo et al. (2023) that were noted to be correlated with performance in drought conditions. The stable QTL for 100 seed weight found on LG 2 and LG 7 were in the same regions as multiple meta-QTL found by Arriagada et al. (2022). Many of the QTL for important traits were in similar regions for both the organically and conventionally bred populations; however, there were exceptions and QTL for several traits were unique to a single breeding history for a particular cross. For example, we identified a marker related to days to 50% flowering in the ORBV-O population on LG 3, but found QTL for the ORBV-C population on LG 1_2 and LG 8_1. Deviations across breeding history such as these were also found for the days to 50% flowering trait in the HYPR populations and for markers related to seed weight per plant. Some populations had significant QTL in different regions depending on the growing system. For example, the HYPR-C population had a stable QTL for vigor on LG 7 in both organic and conventional systems in 2020 and 2021, but there were unique QTL on LG 4 and LG 6–1 that were only significant in the organic growing system in 2020. Similarly, in 2020, there was a significant QTL on LG 7 in both production systems related to days to germination, and a QTL on LG 8 in both systems in 2021. Yet, in both 2020 and 2021, ORBV-O had a unique QTL in the conventional system – one on LG 4 in 2020 and one on LG 2_1 in 2021. For some traits, the populations only had significant QTL in one growing system. For example, there were only significant QTL in the conventional growing system for ‘100 seed weight’, and in the organic system for ‘Basal Root Diameter 1 and 2.’ Environment-specific QTL and distortion are consistent with studies on other crops, some of which suggest that the same traits selected in different environments may actually be different traits resulting from certain genes, or gene networks, activating or inactivating due to environmental signals (Ceccarelli and Grando, 2007; Lin and Togashi, 2002; Pswarayi et al., 2008).

Preliminary work with our data looking at broad-scale genomic differences suggested that populations from the same cross are quite similar at a broad level. Following this, we investigated segregation distortion to determine whether certain chromosomal regions showed differential selection depending on breeding history, and whether there were regions distorted in both crosses. Segregation distortion is common in many plants, including *Phaseolus vulgaris*, and may be linked to incompatibility genes or genes related to sporophytic selection (Blair et al., 2003; Córdoba et al., 2010; Wang et al., 2005). Incompatibility between gene pools is common in beans and may explain some of these selection pressures, although distortion was higher in the HYPR populations. Notably, the HYPR population parents are from more similar gene pools than the parents of the ORBV populations.

In both crosses we utilized, there were significant differences in the SNPs and regions that were distorted in each system (**Figures 5A** and 5B; **Supplementary Tables 11** and **Supplementary Table 12**). Among all sampled bi-allelic SNPs, 9% were skewed in ORBV-O, 8% in ORBV-C, 11% in HYPR-O, and 15% in HYPR-C (**Supplementary Table 12**). Differences between populations from the same cross were compared by linkage group in **Supplementary Table 12** and noted in results. These differences were likely caused by selection in the respective systems, as they came from the same F_1_ populations. There were a few notable regions that had high levels of segregation distortion in all populations. There was a region of high segregation distortion on LG 2 (47–49 Mbp) in all populations, with the exception of ORBV-C, which had some distorted SNPs near this region. In the HYPR populations, there was a strong skew towards the maternal allele in both populations – with a stronger skew in the organic population. In contrast, the paternal allele was favored in this region in the ORBV populations. There were no significant QTL found in this region on LG 2; however, there was a QTL found for root disease in ORBV-C in the organic growing system in 2021 that was skewed towards the maternal allele. All populations had many distorted SNPs that skewed strongly towards the paternal allele on LG 7, along with many distorted SNPs skewed towards the maternal allele on LG 10.

To our knowledge, this work comprises the first combined look at the role of parentage and breeding history on bean performance in organic production systems. Our findings suggest that breeding within organic environments is an effective, and in some cases preferable, approach for harnessing qualitative fitness. We found significant crossover G × E interactions in seedling vigor and root rot disease, indicating breeding under organic management can significantly improve progeny performance in those environments. We also identified important shifts in germination speed and rate within the white-seeded families of organically-bred populations, along with overlapping QTL on linkage groups 4 and 7 for these traits. These QTL may serve as a basis for marker-assisted selection for early-season performance in future organic bean breeding efforts. Although our work identified significant variation within the white-seeded populations, a specific explanation for the higher germination rate in the organically-bred families remains elusive. Further explorations of the seed coat morphology in the white-seeded families of these populations may provide insight into these changes, and how they may best be leveraged in subsequent breeding work. While we were able to improve the linkage maps for our study populations compared to preliminary maps generated by King (2019), the final marker number in the maps remained low compared to similar studies (Bassett et al., 2021; Heilig et al., 2017). This may be attributed, in part, to the relatively small population size of approximately 90 families. Furthermore, we anticipate the low marker number in the HYPR populations is influenced by the genetic relatedness of the parents, which both originate primarily from the Andean background. Vargas et al. (2021) encountered similar issues when SNP genotyping populations generated from parents of the Andean gene pool, although our final marker numbers were higher than their final maps. Given our small population sizes, our work may be improved by using a genotyping-by-sequencing (GBS) approach to generate maps with higher marker numbers.

Broadly, our results suggest that enhanced performance in organic production systems may be captured through breeding and selection within organic production environments. This work led to the identification of specific traits relevant to establishing vigorous and adaptable plants for organic systems. Furthermore, we identified genetic markers associated with these traits, comprising a toolbox of loci that are useful for organic bean breeding. If breeders are able to improve and release varieties that are better suited to organic production, it is reasonable to expect declines in the organic-conventional yield gap. This work has critical implications for the organic industry, as seed companies seek to improve the cultivars that are available for organic production and farmers seek suitable varieties that are competitive in organic environments.

## Data Availability

Data and R code for all analyses and figures are available in a public repository on GitHub (https://github.com/hpark37/SnapBean_BreedingHistory). The SNP genotyping datasetsgenerated for each of the four populations used in this study can be found in the Oregon StateUniversity Scholars Archive at https://ir.library.oregonstate.edu/concern/datasets/rx914010m.

## Supplementary material

Supplementary Figure 1 Linkage maps of organically bred *Phaseolus vulgaris* RIL population, ORBV-O (OR5630 × Black Valentine, organic breeding history). SNP names (consisting of linkage group, physical position in base pairs and base pair substitutions) are shown on the right with corresponding linkage position (cM) shown on the left of each chromosome.

Supplementary Figure 2 Linkage maps of conventionally bred *Phaseolus vulgaris* RIL population, ORBV-C (OR5630 × Black Valentine, conventional breeding history). SNP names (consisting of linkage group, physical position in base pairs and base pair substitutions) are shown on the right with corresponding linkage position (cM) shown on the left of each chromosome.

Supplementary Figure 3 Linkage maps of organically bred *Phaseolus vulgaris* RIL population, HYPR-O (Hystyle × Provider, organic breeding history). SNP names (consisting of linkage group, physical position in base pairs and base pair substitutions) are shown on the right with corresponding linkage position (cM) shown on the left of each chromosome.

Supplementary Figure 4 Linkage maps of conventionally bred *Phaseolus vulgaris* RIL population, HYPR-C (Hystyle × Provider, conventional breeding history). SNP names (consisting of linkage group, physical position in base pairs and base pair substitutions) are shown on the right with corresponding linkage position (cM) shown on the left of each chromosome.
