# Supplementary figures and images for: Horticultural performance and QTL mapping of snap bean (Phaseolus vulgaris L.) populations with organic and conventional breeding histories
Source: Front Plant Sci. 2025 May 19;16:1533039. doi: 10.3389/fpls.2025.1533039 (PMC12177468; doi:10.3389/fpls.2025.1533039)

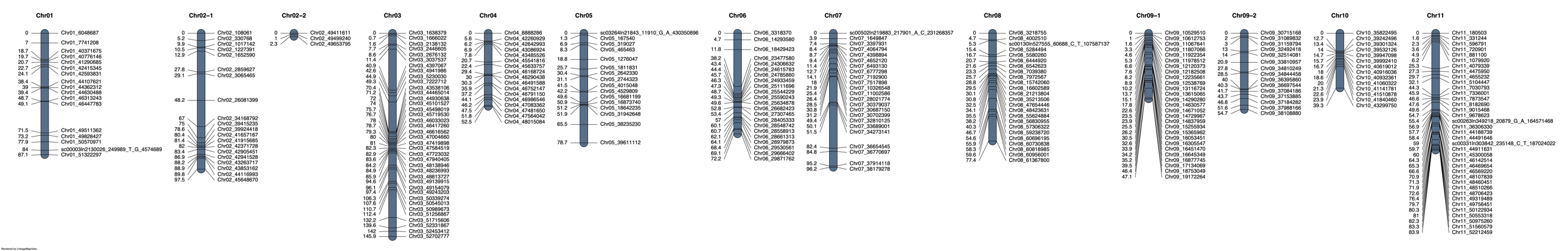

Supplement: Supplementary Figure 1 — Linkage maps of organically bred Phaseolus vulgaris RIL population, ORBV-O (OR5630 × Black Valentine, organic breeding history). SNP names (consisting of linkage group, physical position in base pairs and base pair substitutions) are shown on the right with corresponding linkage position (cM) shown on the left of each chromosome. [file Image1.jpeg]

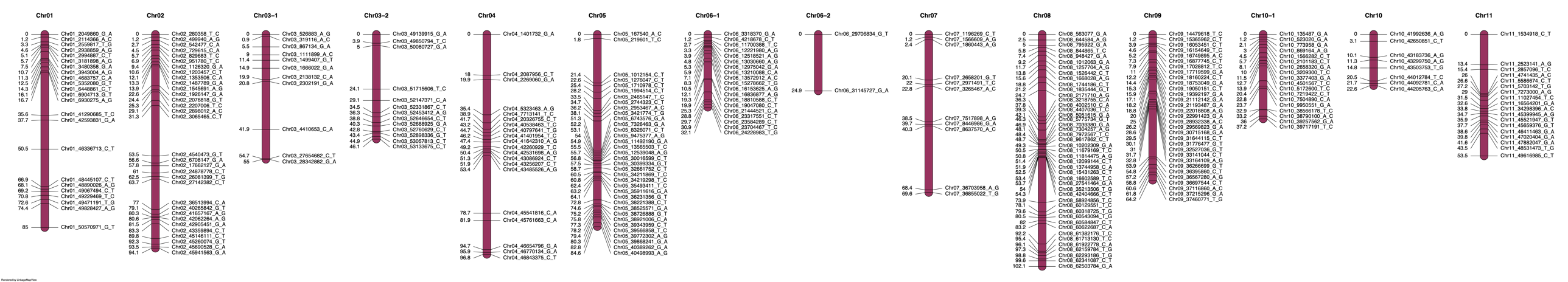

Supplement: Supplementary Figure 3 — Linkage maps of organically bred Phaseolus vulgaris RIL population, HYPR-O (Hystyle × Provider, organic breeding history). SNP names (consisting of linkage group, physical position in base pairs and base pair substitutions) are shown on the right with corresponding linkage position (cM) shown on the left of each chromosome. [file Image3.jpeg]

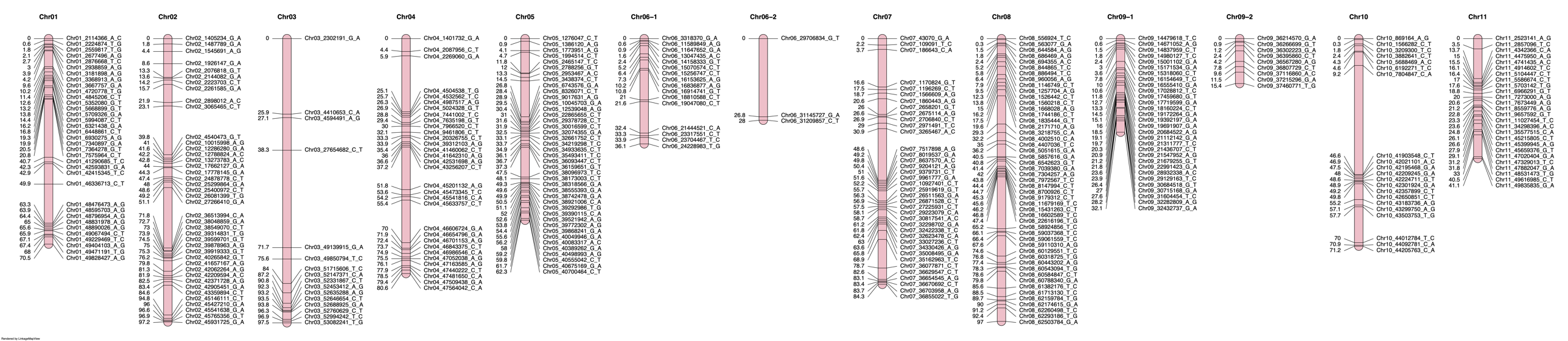

Supplement: Supplementary Figure 4 — Linkage maps of conventionally bred Phaseolus vulgaris RIL population, HYPR-C (Hystyle × Provider, conventional breeding history). SNP names (consisting of linkage group, physical position in base pairs and base pair substitutions) are shown on the right with corresponding linkage position (cM) shown on the left of each chromosome. [file Image4.jpeg]
